# Melioidosis Pneumonia in Saudi Arabia: A Rare Case Report and Review of the Literature

**DOI:** 10.7759/cureus.21871

**Published:** 2022-02-03

**Authors:** Hassan Almarhabi, Adeeb Munshi, Mohammed Althobaiti, Sara Aljohani, Raneen Abu Shanab, Abdulhakeem Althaqafi

**Affiliations:** 1 Infectious Diseases, King Abdullah Medical City, Jeddah, SAU; 2 Infectious Diseases, King Saud Bin Abdulaziz University for Health Sciences, Jeddah, SAU; 3 Infectious Diseases, King Abdullah International Medical Research Center, Jeddah, SAU; 4 Infectious Diseases, King Abdulaziz Medical City, Jeddah, SAU; 5 Radiology, King Abdulaziz Medical City, Jeddah, SAU; 6 Radiology, King Saud Bin Abdulaziz University for Health Sciences, Jeddah, SAU; 7 Radiology, King Abdullah International Medical Research Center, Jeddah, SAU; 8 Internal Medicine, King Saud Bin Abdulaziz University for Health Sciences College of Medicine, Jeddah, SAU; 9 Medicine/Infectious Diseases, King Abdullah International Medical Research Center, Jeddah, SAU

**Keywords:** endemic, south east asia, saudi arabia, burkholderia pseudomallei, melioidosis

## Abstract

Melioidosis is a serious infection caused by the bacterium *Burkholderia pseudomallei (B. pseudomallei)* mostly found in endemic areas like Southeast Asia and Northern Australia. However, in non-endemic regions, such as Saudi Arabia, it remains somewhat rare and unknown to healthcare workers and the public. Herein, we present a case of melioidosis in a 59-year-old Bangladeshi man who presented with pneumonia. He recently returned from Bangladesh, was a known case of type 2 diabetes on metformin, and presented to the emergency department (ED) with a history of cough, shortness of breath, and fever for three weeks. He was initially misdiagnosed and treated as pulmonary tuberculosis in another hospital prior to his latest presentation. Melioidosis is a severe infection that can be misdiagnosed due to variable presentation and low awareness among healthcare workers of the disease. Diagnosis requires high clinical suspicion, especially in patients who are coming from endemic areas with appropriate risk factors such as diabetes mellitus. Treatment with appropriate antibiotics for a long duration, and outpatient follow-up is vital to reduce the risk of recurrence.

## Introduction

The saprophytic environmental gram-negative bacterium *Burkholderia pseudomallei (B. pseudomallei)* is the etiological agent causing melioidosis infection [1]. This motile aerobic bacterium is found mainly in damp soil, unchlorinated water, and plants of tropical regions [1]. Although children are susceptible to being diseased, human melioidosis is an adult infection with a peak incidence at 40-60 years of age with a varied range of severity depending on host risk factors and environmental and strain-specific virulence factors [2].

From bacterial exposure, melioidosis results in acute infection, and in the majority of cases, it presents with sepsis, which can also cause organ failure [3]. A 20-year-based prospective Australian case study reveals that chronic melioidosis (defined as persistent symptoms more than two months) accounts for 11% of the studied population [4]. *B. pseudomallei* has the ability to survive in extreme conditions even without nutrients or even in desert environments. In 2015, its whole genome was sequenced using an air-filtered quantitative polymerase chain reaction (PCR) [5-6].

*B. pseudomallei* infects the body by entering and replicating in epithelial mucosal cell surfaces from any route and spreading their different cell types. The major route for the acquisition of *B. pseudomallei* is through inoculation and inhalation [7-8]. *B. pseudomallei *has the ability to invade the lungs and cause pneumonia [9-10].

*B. pseudomallei* has the ability to remain in latent form for many years and instantly become reactive in the late stage. However, in some cases, it becomes reactive immediately after the exposure and causes many immunological responses [11]. Melioidosis pneumonia is a diverse illness that can be acute with fulminant sepsis and can also cause multifocal lung infiltrates that lead to chronic infection, which mimics tuberculosis both radiologically and clinically [12].

There is a strong correlation between rainfall and melioidosis cases, which is why most cases occur during the wet season [13]. Rainfall or severe storms are directly linked with a shorter incubation period and a higher risk of melioidosis pneumonia [14].

Herein, we present a case of melioidosis in a 59-year-old, Bangladeshi man, who presented with pneumonia in Saudi Arabia.

## Case presentation

A 59-year-old Bangladeshi man, a construction worker, recently returned from Bangladesh, a known case of type 2 diabetes (hemoglobin A1C of 7%) on metformin, presented to the emergency department (ED) with a history of cough, shortness of breath, and fever for three weeks. Ten days prior to his presentation, he was admitted to a private hospital with the same presentation and was managed as pulmonary tuberculosis. He was discharged one day prior to his presentation to our hospital with no improvement and was instructed to isolate himself at home. He denied any history of night sweats, weight loss, or contact with sick patients.

On examination in the ED, the patient was confused, agitated, and severely distressed. He was febrile with a temperature of 38-degree Celsius, respiratory rate was 50 breaths per minute, heart rate was 145 beats per minute, and oxygen saturation of 88% while on ambient air. Respiratory examination revealed abnormal bronchial breath sounds with bilateral crepitations. His abdomen was diffusely tender with no rigidity. The patient was moving all limbs with no evidence of sensory or motor deficit. Other systemic examinations were unremarkable.

Laboratory investigations revealed a high white blood cell count (WBC) (14.5x10^9/L), an increase in neutrophil count (12.69x10^9/L), and normal hemoglobin and platelets. The liver function test showed albumin of 23 g/L, alanine aminotransferase (ALT) of 102 U/L, aspartate aminotransferase (AST) of 67 U/L, normal gamma-glutamyl transferase (GGT), and alkaline phosphatase (ALP). Pancreatic enzymes revealed high lipase (726 U/L) and high amylase (211 U/L). His renal function test was impaired with blood urea nitrogen (BUN) of 27.8 mmol/L and creatinine of 212 umol/L. The patient was found to have low serum PH (7.2), low bicarbonate level (10 mmol/L), high serum ketones (3.8 mmol/L), high serum osmolality (351 mOsmol/kg), and elevated random glucose (31.6 mmol/L). Serology tests for hepatitis B, C, and HIV came back negative (Table 1).

**Table 1:** Investigations of the patient upon admission

Variable	Reference Range	Upon Admission
Hemoglobin (g/dl)	13.0 - 18.0	12.4
Hematocrit (%)	40 - 54.0	40.9
White-cell count	4.0 - 11.0 x10^9^/L	14.5
Neutrophils	2 – 7.5 x10^9^/L	9
Lymphocytes	1.5 – 4 x10^9^/L	1
Sodium (mmol/liter)	135 - 144	146
Potassium (mmol/liter)	3.5 - 4.9	2.5
Chloride (mmol/liter)	101 - 111	112
Bicarbonate (mmol/liter)	22 - 29	10
Urea nitrogen (mmol/liter)	2.8 - 7.4	27.8
Creatinine (umol/liter)	65 - 112	212
Glucose (mmol/liter)	2.9 - 7.8	31.6
Bilirubin, total (umol/liter)	3.4 - 22.1	9.9
Total protein (g/dl)	66 - 83	66
Albumin (g/dl)	39 - 50	23
Alanine aminotransferase (IU/liter)	7 - 44	102
Aspartate aminotransferase (IU/liter)	5 - 34	67
Lactic acid (mmol/liter)	0.7 - 2.0	3.56
Lactate dehydrogenase (IU/liter)	100 - 218	711
Osmolality (mOsm/kg)	288 - 298	351

**Figure 1:**
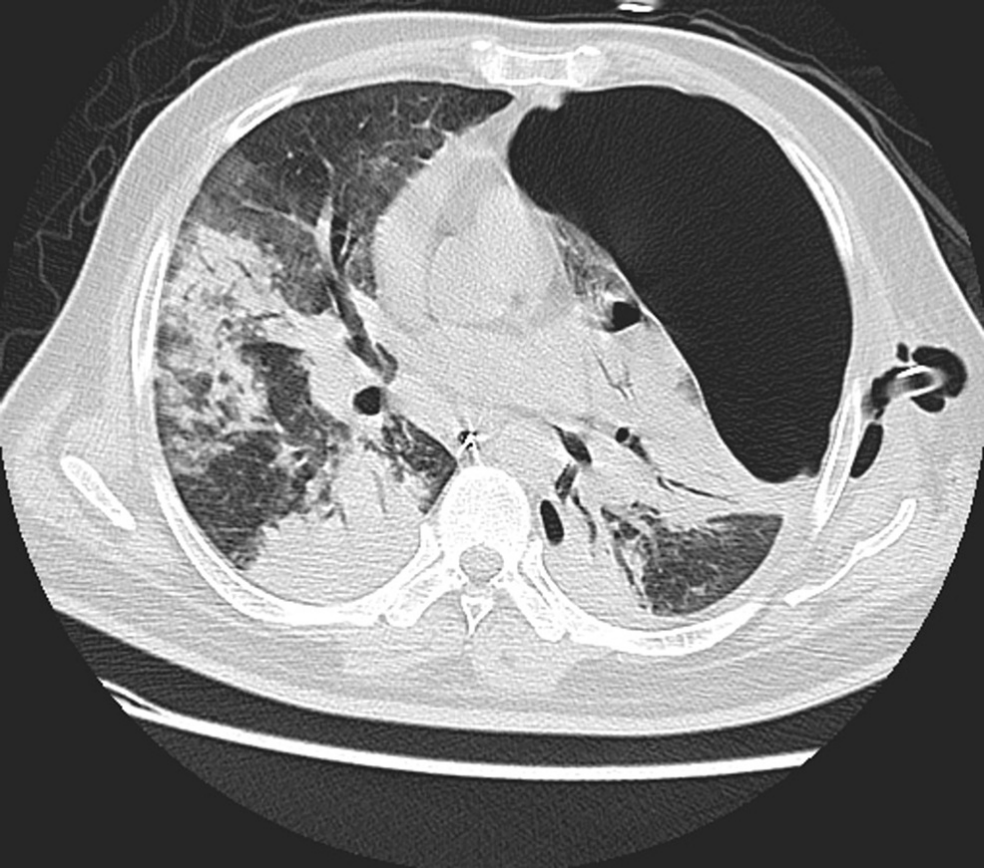
Selected axial CT image showing extensive bilateral consolidation and large left pneumothorax

The patient was admitted as a case of diabetic ketoacidosis (DKA) with acute respiratory distress syndrome (ARDS). The patient was admitted to the intensive care unit (ICU), where he was intubated and put on mechanical ventilation. He was started empirically on rifampicin, isoniazid, pyrazinamide, and ethambutol (antitubercular medications) in addition to linezolid, piperacillin-tazobactam, and doxycycline for the possibility of pneumonia in his situation. Chest X-ray showed bilateral extensive consolidations and left large pneumothorax. Computed tomography (CT) scan of the chest showed extensive bilateral consolidations with a large left pneumothorax (Figure 1).

**Figure 2:**
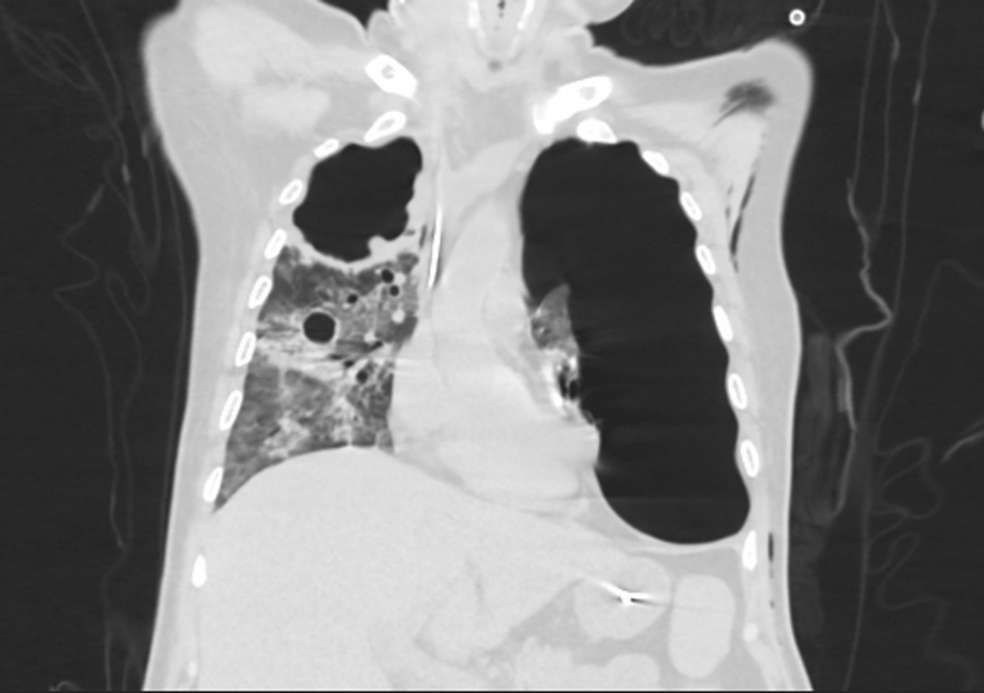
Selected coronal CT image showing a large right upper lobe cavity with an irregular nodular wall

On the second day of his admission, he developed a left pneumothorax, and a chest drain was inserted. Analysis of pleural fluids revealed a PH of 6.53 and pleural fluid lactate dehydrogenase (LDH) of 16872 U/L (Table 2). Two days later, respiratory and pleural fluids showed gram-negative bacilli, which were later identified using the VITEK® MS (bioMérieux Clinical Diagnostics, Durham, NC) as *B. pseudomallei*, which was susceptible to ceftazidime, meropenem, and trimethoprim-sulfamethoxazole. On the other hand, it was resistant to gentamicin and amikacin. The patient was critically ill in the ICU and was on maximum ventilatory support; antibiotics were changed to meropenem and trimethoprim-sulfamethoxazole. As three, consecutive, respiratory acid-fast bacilli smears were negative, anti-tuberculosis medications were stopped. The patient slowly improved until he could be discharged to the general medical ward. On Day 18 of hospitalization, he spiked a fever of 39°C. Sputum culture was taken, which showed *Aspergillus fumigatus*. Serum galactomannan was taken as well, which revealed positive results of 0.65. A CT scan of the chest showed a large right upper lobe cavity with an irregular nodular wall suggestive of fungal infection, which was not found in the initial imaging (Figure 2).

**Table 2:** Results of the pleural fluid analysis

Pleural fluid	Result
PH	6.5
Lactate dehydrogenase (IU/liter)	16872
Protein	6.53
Glucose	16.8

The patient was started on voriconazole 200 mg twice a day, meropenem 1 g three times a day, and trimethoprim-sulfamethoxazole 320 mg twice a day. The patient significantly improved on this regimen. He received 55 days of meropenem and trimethoprim-sulfamethoxazole in addition to 35 days of voriconazole. He was discharged on oral trimethoprim-sulfamethoxazole for another six months and voriconazole for another two months. The patient was asked to follow up with the infectious disease team, but, unfortunately, he was lost to follow-up.

## Discussion

It is well-established that melioidosis is a serious and possibly life-threatening infection in endemic areas like Southeast Asia and Northern Australia. However, in non-endemic regions, such as Saudi Arabia, it remains somewhat rare and unknown to healthcare workers and the public [15-16]. Thailand reported the highest number of infections, with approximately 2000-3000 cases each year. Cases of melioidosis pneumonia are found around the globe, predominantly between 20 degrees North and 20 degrees South. It is mostly exposed during the monsoon season [4-17]. 

To the best of our knowledge, our case is the third reported case of confirmed melioidosis in Saudi Arabia. Alwarthan et al. reported a case of a provisional diagnosis of melioidosis, unfortunately, the definitive diagnosis could not be confirmed due to a lack of facility at their laboratory [16]. Alhatmi et al. reported two cases of Saudi patients, who recently returned from an endemic area, with confirmed melioidosis by isolating gram-negative bacilli in the blood culture, which was later identified using an automated machine VITEK as *B. pseudomallei* [18]. The United Arab Emirates, Egypt, and Turkey have reported a small number of cases as well [15].

This lack of awareness among physicians and microbiologists could have resulted in underreporting melioidosis cases [1]. Risk factors for melioidosis include diabetes, chronic lung, liver, and kidney diseases, excessive alcohol consumption, and malignancy. Diabetes mellitus is the most common risk factor, which imposes a 12-fold risk increase for melioidosis, as 23-60% of diagnosed patients are diabetic [1-3]. Chronic kidney disease was the sole factor associated with higher mortality since patients experience the highest rate of bacteremia [9]. That explains our patient’s complicated condition because he was diabetic.

The incubation period of melioidosis can vary from a few days to months or years before developing symptoms [11]. Its presentation can vary from asymptomatic infection, acute localized infection, or septicemia, to chronic infection [19]. An acute infection accounts for 85% of melioidosis cases, where a pulmonary infection is the most commonly reported presentation combined with bacteremia in adults, with symptoms including fever, cough, pleurisy, and night sweats [1-2]. However, it can occur in chronic or recurrent matter approximately 11% and 4%, respectively [2]. A few patients suffer from complicated respiratory infections and disseminated lung infections. Empyema, consolidation of one or more lobes, and nodule formation in the pleural cavity are common features of lung and pleural melioidosis [20].

Many melioidosis cases are misdiagnosed due to variable non-specific manifestations of the disease that can mimic others such as tuberculosis and community-acquired pneumonia [1-2]. This is similar to the present case where the patient was falsely diagnosed and treated for TB before confirming the diagnosis of melioidosis with multiple positive cultures. Additionally, when the differential is indicated, it is necessary to consider *Burkholderia pseudomallei*, mainly because it is resistant to many antibiotics commonly used empirically, including ampicillin, second-generation cephalosporins, macrolides, rifampicin, and aminoglycosides [21].

The diagnosis of melioidosis can be challenging, especially when the disease occurs outside an endemic region [22]. It can be confirmed by a positive culture of the organism from any region of the body [23]. A thorough screening of the patient’s blood, sputum, urine, pus culture, and throat swab should be done as well [16]. The sensitivity and specificity of direct immunofluorescence microscopy of samples taken from infected sites, such as sputum, urine, and pus, is 70% and 98%, respectively [16]. It gives an advantage of rapid diagnosis within 30 minutes, which can be helpful in initiating the treatment [24]. In addition to direct immunofluorescence microscopy, a polymerase chain reaction is another tool, which can be helpful for rapid diagnosis [24]. The most sensitive test for diagnosing melioidosis is the indirect hemagglutination assay. However, it is not helpful when the diagnosis is suspected in endemic regions, as around 50% of patients might yield positive results. Nevertheless, a titer higher than 1:640 is indicative of the diagnosis [22]. Chest radiograph, computed tomography (CT), and ultrasound are extremely helpful to determine the extent of the disease as well as the presence of abscesses [22].

More appropriate antibiotics like ceftazidime and carbapenems were effective in reducing mortality rates by up to 50% in severe melioidosis [25]. Treatment of melioidosis consists of two phases, which are the intensive and eradication phases. In the intensive phase of treatment, ceftazidime or carbapenems with or without trimethoprim-sulfamethoxazole for at least 14 days is the treatment of choice [19]. Trimethoprim-sulfamethoxazole with or without doxycycline for a minimum duration of 12 weeks is used in the setting of the eradication phase. Treatment can be extended beyond these durations, that is four weeks for the intensive phase and 20 weeks for the eradication phase [13]. Luckily, our patient improved when he was started on the right antibiotics despite his severe and complicated infection. Reported mortality rates of melioidosis are worryingly high, ranging from 19-40%, with a sharp increase in cases of septic shock (>80%) [26]. Therefore, physicians need to have a low threshold of suspicion, be aware of the clinical presentation of melioidosis, and diagnose it as early as possible, especially when patients have suggestive presentation [27].

## Conclusions

Melioidosis is a severe infection that can be misdiagnosed due to variable presentation and low awareness of the disease among healthcare workers. Diagnosis requires high clinical suspicion, especially in patients who are coming from endemic areas with appropriate risk factors such as diabetes mellitus. Treatment with appropriate antibiotics for a long duration and outpatient follow-up is vital to reduce the risk of recurrence.
